# Editorial: The interpersonal effects of emotions: The influence of facial expressions on social interactions

**DOI:** 10.3389/fpsyg.2022.1074216

**Published:** 2022-11-08

**Authors:** Laura Sagliano, Marta Ponari, Massimiliano Conson, Luigi Trojano

**Affiliations:** ^1^Department of Psychology, University of Campania Luigi Vanvitelli, Caserta, Italy; ^2^Independent Researcher, Barcelona, Spain

**Keywords:** social judgement, emotional facial expression, facial expressions, emotion, emotion recognition

Facial expressions convey information about one's emotional state and are one of the most important forms of non-verbal communication. Facial expressions also represent a powerful factor affecting other individuals' affect, cognition, and behavior (van Kleef and Côté, 2022). The present topic is aimed to gather studies addressing the issue of facial expressions processing from different perspectives. The main lines of investigation pursued here aim to clarify which conditions (e.g., awareness, neurodegenerative disorders) could affect recognition and comprehension of facial expressions, and to further our understanding on how facial expressions affect cognitive processes in different contexts (e.g. risk decision, social judgement).

When encountering someone who expresses an emotion, individuals tend to mimic that facial expression automatically (Hatfield et al., 1993; Dezecache et al., 2013). This phenomenon is an aspect of emotional contagion, which is related to multiple processes implied in sharing emotional states between two or more persons. Namba et al. investigated the processes responsible for elicitation and sharing of emotions by means of a computational model (multinomial processing tree). Their findings indicate that emotional valence exerts a key role in modulating emotional sharing: while both happy and angry facial expressions evoke an emotional state, only happy expressions elicit emotional sharing in the perceiver; instead, the perceiver can appreciate the negative valence of anger expressions, but rarely shares it.

Previous studies (e.g., Anderson et al., 2012; Sagliano et al., 2020) suggested that observer's judgements about the social context can be influenced by facial expressions even when these are not consciously perceived. However, whether conscious perception is a prerequisite for attention to be captured by fearful expressions is still a matter of debate. Baier et al. collected both psychophysiological and behavioral data in participants who were required to search for neutral face targets on a display; invisible (backward-masked) or visible fearful faces were used as cues. The findings demonstrate that participants' performance was not affected by invisible fearful faces, suggesting that awareness is a prerequisite for attention capture by facial expressions.

Because of the relevance of facial expression recognition for social interactions and survival, clarifying what factors could increase or interfere with their recognition is crucial. An et al. assessed whether emotional congruency and similarity can enhance face-context associative memory in healthy participants. The authors manipulated the emotional similarity between facial expressions (i.e., disgust, anger, and fear) and the context they were presented into. The results showed that both face memory and face-context associative memory benefited from emotional congruency and similarity. Indeed, the face memory and the face- context associative memory were the greatest in the congruent condition, intermediate in the high similarity condition, and worst in the low similarity condition. These findings supported the idea that both facial expressions and emotional contexts can affect an individual's memory in everyday social interactions.

Another important aspect to understand is whether the processing of facial expressions is prioritized over other facial features (e.g., age and ethnicity; Weisbuch and Ambady, 2008; Kozlik and Fischer, 2020). Wentura and Paulus investigated the way individuals process facial expressions and ethnicity (group membership) to understand whether these facial features are extracted independentely of each other or are immediately integrated. The authors employed an approach/avoidance task with either happy/fearful faces of German and Turk individuals or happy/fearful faces of young and old persons, as previous literature (Kite and Johnson, 1988; Wagner et al., 2003) revealed that in Germany there are prejudices against Turk/Middle-eastern persons as well as against the elderly. Overall, their findings support the idea that information about emotion and group membership is immediately integrated in social interactions, consistent with the social message account hypothesis (Weisbuch and Ambady, 2008).

As social signal, facial expressions could also affect decision making (Averbeck and Duchaine, 2009). In a study with four experiments assessing different implementation of risks, payoffs, probabilities, and temporal decision requirements, Winkielman et al. demonstrated that emotional facial expressions presented implicitly influenced in different ways participants' attitude toward risk: positive expressions increased risk seeking whereas negative expressions increased risk aversion. These findings support the idea that judgment and decision making are based not only on cognitive factors but also on the analysis of social cues such as facial expression.

The effects of facial expressions on social judgement has been assessed by Ueda and Yoshikawa in three experiments. Healthy participants were required to judge from a third person perspective which of two persons (facing each other) was more appropriate for forming alliances, more trustworthy, or more attractive. The facial expression of the character was manipulated (i.e., happy, sad or angry expressions). Their findings demonstrate that at short presentation durations individuals were more likely to choose persons with happy faces than those with other expressions. On the contrary, at longer presentation times, facial expressions did not affect participants' judgments. Thus, facial expressions could affect individuals' judgement when there is not enough time to elaborate their appraisal, suggesting that time of processing is a key factor for processing of social information.

From a clinical neuropsychological viewpoint, one paper of this topic addressed emotion recognition in Parkinson's disease (PD). As in other neurodegenerative disorders, patients with PD often show deficits in emotion recognition (for a meta-analysis, see Coundouris et al., 2019), and in attributing affective or cognitive mental states to others (theory of mind; ToM; e.g., Maggi et al., 2022). Dodich et al. assessed emotion recognition, affective and cognitive ToM in cognitively unimpaired or impaired (i.e., with mild cognitive impairment; MCI) patients with PD. The overall results showed that patients performed worse than age-matched healthy controls on emotion and intention attribution tasks. Moreover, patients with PD-MCI achieved significantly lower scores than both the control group and the cognitively unimpaired patients on the facial expression recognition task, especially for negative emotions. These results suggested that emotional processing could be an early marker of cognitive decline affecting social interactions in PD.

In line with the aim of the topic, these studies contributed to the advance of knowledge about the interpersonal consequences of facial expressions employing different paradigms and methodological approaches. More importantly, these studies raised interesting questions to be addressed in future research.

## Author contributions

All authors listed have made a substantial, direct, and intellectual contribution to the work and approved it for publication.

## Conflict of interest

The authors declare that the research was conducted in the absence of any commercial or financial relationships that could be construed as a potential conflict of interest.

## Publisher's note

All claims expressed in this article are solely those of the authors and do not necessarily represent those of their affiliated organizations, or those of the publisher, the editors and the reviewers. Any product that may be evaluated in this article, or claim that may be made by its manufacturer, is not guaranteed or endorsed by the publisher.
